# Addressing barriers of community participation and access to mass drug administration for lymphatic filariasis elimination in Coastal Kenya using a participatory approach

**DOI:** 10.1371/journal.pntd.0008499

**Published:** 2020-09-16

**Authors:** Doris W. Njomo, Lydiah W. Kibe, Bridget W. Kimani, Collins Okoyo, Wyckliff P. Omondi, Hadley M. Sultani

**Affiliations:** 1 Eastern & Southern Africa Centre of International Parasite Control (ESACIPAC), Kenya Medical Research Institute (KEMRI), Nairobi, Kenya; 2 Division of Vector Borne and Neglected Tropical Diseases, Ministry of Health, Nairobi, Kenya; Erasmus MC, NETHERLANDS

## Abstract

Since the prioritization of Lymphatic Filariasis (LF) elimination in 1997, progress has been made in reducing disease transmission and burden. Validation of elimination through Transmission Assessment Surveys (TAS) in implementation units (IUs) that have received at least 5 rounds of mass drug administration (MDA) and achieved minimum threshold of 65% treatment coverage is required. There are IUs that do not qualify for TAS due to achievement of low treatment coverage. This study sought to identify barriers of community participation and access to MDA, develop and test strategies to be recommended for improved uptake. Two wards in Kaloleni sub-county, Kilifi county with an average treatment coverage of 56% in 2015, 50.5% in 2016 were purposively sampled and a quasi-experimental study conducted. Through systematic random sampling, 350 (pre-intervention) and 338 (post-intervention) household heads were selected and interviewed for quantitative data. For qualitative data, 16 Focus Group Discussions (FGDs) with purposively selected community groups were conducted. Participatory meetings were held with county stakeholders to agree on strategies for improved community participation in MDA. The quantitative data were analyzed using STATA version 14.1, statistical significance assessed by chi square test and qualitative data by QSR NVIVO version 10. The identified strategies were tested in experimental sites during the 2018 MDA and the usual MDA strategies applied in control sites. The results showed an increase in community participation and access to MDA in both sites 80.6% (pre-intervention), 82.9% (post-intervention). The proportion of participants who considered the treatment as necessary significantly (p = 0.001) increased to 96.2% from 88.3% and significantly dropped for those with drug swallowing problems associated with: size (p<0.001), number (p<0.027) and taste (p = 0.001). The implemented strategies may have contributed to increased participation and access to MDA and should be applied for improved treatment uptake. Health education on disease aetiology and importance of drug uptake in all rounds is key to program’s success.

## Author summary

Elephantiasis is targeted for elimination according to World Health Assembly resolution of 1997. There is need to determine if spread of infection has been stopped by conducting evaluations in areas that have received at least 5 rounds of treatment at minimum levels of 65% coverage. There are areas that cannot qualify for evaluations due to their achievement of low coverage. We conducted a study in Kaloleni sub-county to identify barriers of community participation in the campaign, develop and test strategies for improved treatment uptake for recommendation to the Programme Implementers. The study involved collecting quantitative data from systematically randomly sampled household heads and qualitative data from purposively selected key stakeholders. The identified strategies were tested in experimental sites while the usual treatment strategies were applied in control sites. The results showed an increase in community participation in MDA and in persons who considered the treatment as necessary. The strategies may have contributed to decreased proportions of persons who expressed problems with drugs swallowing due to size, number and taste, to positive perceptions of the drugs and should be applied for improved treatment uptake. The communities need robust health education on elephantiasis and importance of consistency in drug uptake.

## Introduction

The Global Programme to Eliminate Lymphatic Filariasis (GPELF) was launched over 15 years ago with the goal to interrupt transmission of the disease in endemic countries [1]. The GPELF has the aim of interrupting LF transmission, managing morbidity and preventing disability and recommends annual community-wide MDA of antifilarial tablets; diethylcarbamazine citrate (DEC) or ivermecticin and albendazole to entire at-risk populations aged 2 years and above for 4 to 6 years at adequate levels of coverage, at least 65% of total population in endemic areas [2, 3]. Since the prioritization of LF elimination, progress has been made in reducing transmission and burden of disease. At the start of GPELF, 81 countries were considered endemic for LF and previous reports indicated an estimated 46% reduction of the population at risk of infection [4] and cure and prevention of over 96 million cases [5,6]. By 2017, 7.7 billion treatments had been delivered to more than 910 million people at least once in 68 countries, considerably reducing transmission in many places and the population requiring MDA had declined by 42% (597 million) where infection prevalence had been reduced below elimination thresholds [7].

Only China and the Republic of South Korea were declared to have eliminated LF as a public health problem in 2007 and 2008, respectively [8] and Thailand and Egypt submitted their dossier for validation of elimination in 2017 [7]. There is a need to urgently scale up activities in all the countries. There are IUs in the LF endemic countries that have completed at least 5 effective MDA rounds to qualify for TAS in order to evaluate the level of LF transmission in the population and determine if MDA can be stopped [9]. There are however IUs that do not qualify for TAS as they have consistently been achieving low MDA coverage. Fear of side effects, perception of no need for LF drugs and dislike of taking too many tablets [10–12] are some reasons for low MDA coverage.

In Kenya, LF is endemic in 6 counties of the Coastal region and the Ministry of Health launched its LF elimination programme with DEC, 6 mg/kg plus albendazole (400 mg) in 2002 in Kilifi district as the first IU. In 2003, the Programme was scaled up to include Malindi and Kwale districts and in 2005 and 2008, the 3 districts received additional rounds of MDA. Due to technical and administrative challenges, sustained annual MDA was not possible. In 2011 a further round to include Tana River and Lamu counties was conducted. In 2015, an additional round was administered and included Taita Taveta county which was receiving its initial round. In 2016, the Programme was scaled up to include Mombasa county thus achieving 100% geographical coverage and in 2017, 2018 and 2019 MDA was implemented in all 6 counties. An epidemiological survey conducted in 2015 in sentinel sites of Lamu, Tana River, Kilifi, Kwale and Taita Taveta counties showed an overall prevalence circulating filarial antigen (CFA) positive persons of 1.3% with Kilifi and Kwale counties having a prevalence < 1.7% thus justifying a need for additional rounds of MDA in these counties [13].

Community volunteers, known as community drug distributors (CDDs) selected by the community members to deliver drugs to individuals at their homes have been used in all the rounds of treatment. Each CDD is expected to cover a total of 100 households. The sub-county Medical Officer and political authorities are the first to be sensitized on MDA: endemicity of the area; purpose of mass treatment; drugs used; method of distribution; length of distribution and role of WHO in the programme followed by peripheral health providers who then sensitize community leaders. The community leaders through open meetings at community level sensitize community members and together they select CDDs. The CDD selection criteria include: ability to read and write; keep records; trustworthiness; well known by the community members; and willingness to distribute drugs to all eligible persons in allocated areas [14]. The CDDs who are also charged with undertaking the role of community sensitization are trained by health personnel. The distribution of drugs is done house-to-house and the whole exercise takes 5 days: 2 days registration and 3 days drug administration including revisits to those missed out on initial days. The CDDs are given monetary incentives amounting to Kenyan Shillings 500 (5 USD) for each day by the Programme. In the Kenyan health system, community health extension workers (CHEWs) are key stakeholders who support the delivery of community-based health promotion and prevention activities and supervise the distribution as well as help manage any side effects [15].

The ongoing global elimination effort is faced with the challenges of people accepting drugs when they have no symptoms of the disease [16]. No group of persons should be left totally untreated because such groups if infected form reservoirs of microfilariae (mf) contributing to continued transmission of infection [17]. Previous studies conducted in Kenya reported age and migration among urban population and poor community mobilization and sensitization in rural areas as reasons for poor access to treatment [18, 19]. Community participation has been found to be one of the major challenges to the MDA program and its absence is known to hamper successful implementation [20]. Community-based participatory approach is a partnership approach to research that involves community members, organizational representatives, and researchers in aspects of the research process and in which all partners contribute expertise and share decision making and ownership. The aim is to increase knowledge and understanding of a given phenomenon and integrate the knowledge gained with interventions and policy and social change to improve the health and quality of life of community members [21]. The current study was conducted in Kilifi county which had received 8 rounds of MDA and used a community-based participatory approach to identify, develop and test strategies for improving community participation and access to MDA.

## Methods

### Ethics statement

Ethical clearance was received from the Kenya Medical Research Institute (KEMRI), Scientific and Ethics Review Unit (SERU) Protocol Number 3666 and written informed consent sought from all study participants. An information sheet was provided to all individuals 18 years and above invited to participate in the study in Swahili. Participants underwent written informed consent and agreed to have the questionnaire data captured in ODK and the FGDs audio-recorded. During data capture and transcription, participant names were replaced with alphanumeric unique identifiers to ensure anonymity and confidentiality.

### Study area

**Fig 1:**
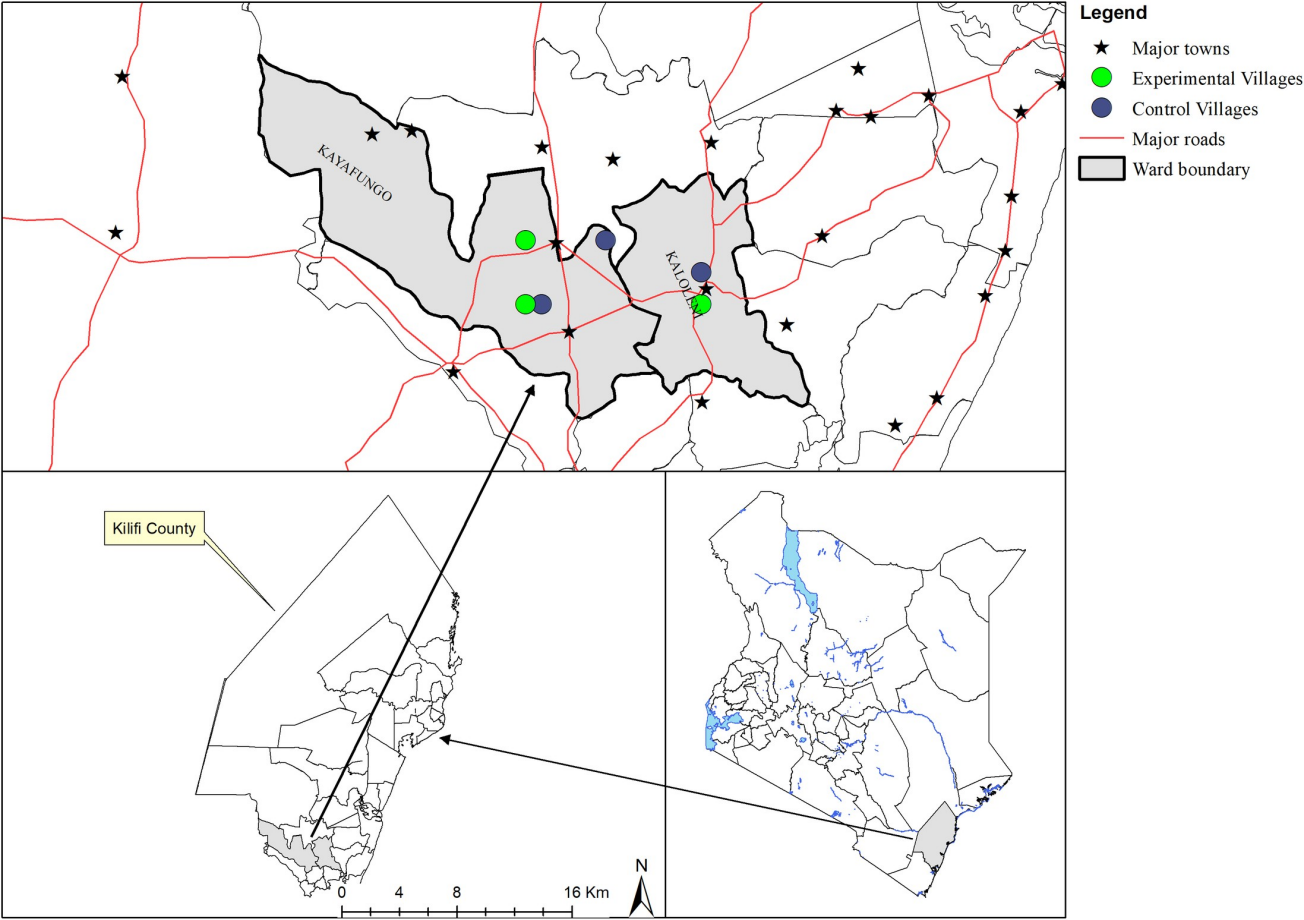
Map of the study area showing the villages.

Kilifi county has a population of 1,109,735 and covers an area of 12,245.90 km^2^ [22]. The county is located north and northeast of Mombasa, the second largest town in Kenya and is administratively made up of 7 sub-counties. The county is endemic for LF caused by *Wuchereria bancrofti* and has a prevalence of filarial antigenaemia of < 1.7% and a mean microfilarial density of < 25 MF/ml [13]. The current study was conducted in Kaloleni sub-county which has a population of 159, 739 [22]. Two wards i.e. Kaloleni with a population of 41, 689 of which an average of 36.8% is urban [22] and Kayafungo with a population of 22, 250 people formed the rural study area (Fig 1). Farming and business are the main economic activities of the area.

### Study design and setting

This was a quasi-experimental study with a pre-intervention and a post-intervention phase that utilized quantitative and qualitative methods for data collection. Based on the 2015 and 2016 MDA Programme data from MOH, Kenya, Kaloleni and Kayafungo wards were selected purposively for the study. In 2015, Kaloleni ward had a treatment coverage of 58% and Kayafungo, 54% and in 2016, Kaloleni had 62% and Kayafungo 39% all below the recommended minimum treatment coverage of 65%.

In the pre-intervention phase, the 2017 MDA was used as a basis of enquiry on the usual MDA process and through a community-based participatory approach, strategies aimed at enhancing uptake were developed. The developed strategies were then tested in selected experimental villages of the 2 wards prior to and during the 2018 MDA while in the control villages the usual MDA strategies were used. Thereafter an impact assessment was conducted to compare differences and similarities in community participation and MDA access between the experimental and the control villages.

### Sampling and study population

In Kaloleni ward, 2 villages were purposively chosen; Vishakani and Town Centre which were assigned as control and experimental villages respectively. In Kayafungo ward 4 villages were purposively chosen; where Gandini A and Gandini B were assigned as control villages while Mirihi ya Kirao and Gogoraruhe were assigned as experimental. In the assignment of control and experimental villages, the study aimed to have urban and rural settings incorporated. Therefore, a total of 6 villages in the two wards were purposively selected with the support of the area chiefs.

### Data collection

The quantitative and qualitative data were collected separately using the concurrent triangulation method to allow for comparison and divergence of views after analysis [23]. For the quantitative arm, the head of each household was selected and a survey questionnaire (S1 Text) administered by trained field assistants using Open Data Kit (ODK), a mobile-based data collection system with in-built quality checks to prevent errors. The questionnaire used to collect data from the consenting household heads included information on socio-demographic and socio-economic characteristics, knowledge about LF and MDA, anti-filarial drug uptake, and their perception of the treatment. The household head was selected to respond to the questionnaire if he/she was an adult of 18 years and above, gave informed consent and had resided in the selected village for the last three years. The quantitative data were collected using house-to-house approach by six teams i.e. one team per village. Each team consisted of one trained field assistant, one community health volunteer (CHV) and a village chairperson. There was one supervisor responsible for all the six teams who ensured that data collected were of high quality and were sent daily to the ODK server, and ensured smooth coordination of the teams and allocation of the households to be visited.

To elicit more information on opportunities for improved community participation and access to MDA, qualitative data was collected in both pre- and post-interventions phases through 16 focus group discussions (FGDs) that were carried out with adult and youth male and female single-sex groups to assess their perceptions and gather inputs for developing strategies which were then tested (S2 Text). The number of FGDs was determined by the level of saturation. The design was iterative and there was a back and forth process which included data collection and analysis and further sample selection therefore giving early insights and influencing selection of more participants up to the point where no newer information was being gathered. Standard procedures including maintaining a neutral stance, probing and allowing the respondents to express themselves without asking leading questions, asking general questions before specific questions and varying questions wording to avoid seeming repetitive were adhered to [24]. Each FGD took 40 to 50 minutes at a quiet and private venue to ensure that there were no disturbances. The data collection was moderated by KEMRI social scientists assisted by trained field assistants using Swahili, the local language. Notes were taken during the FGDs and voice recorders were used to record all the information.

The field assistants were trained on both quantitative and qualitative data collection methods for three days prior to the start of data collection exercise to enable them understand the objective of the study, survey protocol, data collection techniques, and proper use of ODK system, voice recorders and data transcription and translation.

Through participatory meetings with county (S3 Text) and sub-county (S4 Text) stakeholders including governance of health sector, education sector, religious sector, Non-Governmental organization representatives, county commissioner and deputy county commissioners and the local administrative leaders at the wards level, barriers of community participation in the program and opportunities for improved MDA process were identified and intervention measures agreed upon. The meetings were held at county and sub-county commissioners’ boardrooms and chaired by the county LF coordinator supported by the sub-county LF coordinator. The agenda included presentation of the background and objectives of the study as well as the results of the pre-intervention phase by the Principal Investigator. Minutes of the meetings were taken by one of the study’s co-investigators. The presentation formed a basis of discussion for implementable strategies for improved community participation and access to MDA. During the plenary sessions, all stakeholders participated and there was no domination observed from any parties. Generally, the strategies were agreed upon between both genders and across all age groups.

### Data management

The hard copies of the qualitative data were stored in lockable and secure cabinets. The recorded data was coded and later transcribed and translated into English. Double transcription and translation and back translation was done among the investigators so as to agree on the meaning of the transcripts and minimize biasness. To ensure quality control, the soft copies of both quantitative and qualitative data were stored in computers with passwords, with authorized access by the Principal Investigator.

### Statistical analysis

The quantitative data were analyzed using STATA version 14.1 (STATA Corporation, College Station, TX, USA). Participants’ responses were pooled and arranged in different categories. Proportions were calculated for variables of interest and their 95% confidence intervals (CIs) were calculated using generalized linear models that accounted for wards (clusters). Differences in proportions of variables of interest were compared using two-sample proportion tests and Pearson’s chi-square tests where necessary.

Associations between uptake of LF drugs and variables of interest were assessed using Pearson’s chi-square tests. Access to MDA was defined as proportion of participants who reported receiving treatment during the last MDA round. Socio-demographic and economic factors influencing access to MDA were analyzed using univariable analysis and the strength of the association measured as odds ratio (OR) using mixed effects logistic regression at 95% CI. The univariable analysis on factors influencing access to MDA was conducted on all participants both in the control and experimental groups.

The qualitative data were coded and entered into QSR NVIVO version 10 for management and analysis. Manual analysis was further conducted according to study themes which were determined prior to the analyses. A code sheet was created following the FGDs guides after which, the textual data was coded into selected themes and a master sheet analysis was carried out, giving all the responses a theme. Ideas were then formulated by looking at the patterns of responses. A framework approach was adopted based on themes around barriers and facilitators of community participation and access to MDA: communities’ socio-demographic characteristics; knowledge and perceptions of lymphoedema and hydrocele; knowledge and perceptions of the MDA processes to include uptake of MDA, awareness creation and timing of the MDA activity, drugs used and eligibility criteria. The analyzed data was presented in text form.

The data from the two sources was triangulated for cross-validation and corroboration of the findings within the study. This helped to increase the credibility and validity of the results by continuously cross-checking the two data sources for consistency and divergence of views and thus overcoming weakness of one method having strength over the other.

## Results

### Demographics

A total of 350 households from 6 villages were surveyed from two wards for the pre-intervention phase and a total of 338 households for the post-intervention phase which was conducted 7 days after the November 2018 MDA activity. During the implementation phase, the villages were divided into control and experiment groups based on their proximity to one another in an effort to control for spillover effects. Three villages; Vishakani, Gandini A and B constituted the control group while Town Centre, Mirihi ya Kirao and Gogoraruhe formed the experimental group (Table 1).

**Table 1:** Number of households selected in each village.

Ward	Village	No. of households		Village Type
		Pre-intervention phase	Post-intervention phase	Control/ Experimental
	Town Centre	88	82	experimental
Kaloleni	Vishakani	88	87	control
	Mirihi ya Kirao	43	43	experimental
	Gogoraruhe	46	42	experimental
	Gandini A	42	42	control
Kayafungo	Gandini B	43	42	control
Total		350	338	

### Background characteristics of the quantitative arm study participants

The majority of household heads were female 433 (62.9%) with a mean age of 43.1 years (SD = 16.4 years and range 18–88 years). A large proportion, 533 (77.5%) was in marital unions, and slightly more than two-thirds 464 (67.4%) were Christians. Less than a half, 332 (48.3%) had completed primary level of education. Regarding main occupation, about two-fifths, 291 (42.3%) were engaged in farming while 124 (18.0%) were in small businesses and 109 (15.8%) were casual workers.

Socio-economic factors were measured by use of proxy indicators. Observed toilet ownership among the surveyed households was 79.5% with majority of the toilets being traditional pit latrines 454 (83.0%). The flush toilets and ventilated improved pit (VIP) latrines were owned by 12.1% and 4.9% of the surveyed households respectively. Assessment of the household structures showed that most houses had roofs made of iron sheets 539 (78.3%) and others, thatch/palm leaf/makuti 148 (21.5%), most walls were made of mud/dung 415 (60.3%) or bamboo with mud 82 (11.9%), and most floors were made of earth/mud/dung 521 (75.7%) while 130 (18.9%) were made of cement. Majority, 94.2% of the dwellings were owned by the family while only 5.2% were rented. One quarter, 25.3% of the households drew their water from unprotected well, 24.7% from surface water e.g. rivers, dams, ponds or canals, and only 18.8% had water piped in their compounds/plots or access to public taps.

### Background characteristics of the qualitative arm study participants

The single-sex adult and youth male and female FGDs participants included adults (35 years and above) and youth (18 to 34 years) respondents of homogenous characteristics. Over one half (59.4%) of the participants were Christians, 35% had attained primary school level of education while 29.7% were secondary school leavers. Regarding the main occupation, over one third, 34.9% were owners of small businesses and 22.4% were small scale farmers. Each FGD contained a minimum of 8 and a maximum of 12 participants. The FGD quotes are presented under the following codes:

- FGD-KLN-CM-YF-001-012 which stands for Focus Group Discussion- Kaloleni- Community Member- Youth Female and number assigned during the FGD could be 1–12 as per number of participants.
- FGD-KYG-CM-AW-001-012 which stands for Focus Group Discussion- Kayafungo- Community Member- Adult Women and number assigned during the FGD could be 1–12 as per number of participants.
- FGD-KYG-CM-AM-001-012 which stands for Focus Group Discussion- Kayafungo- Community Member- Adult Men and number assigned during the FGD could be 1–12 as per number of participants.
- FGD-KLN-CM-YM-001-012 which stands for Focus Group Discussion- Kaloleni- Community Member- Youth Male and number assigned during the FGD could be 1–12 as per number of participants.

The intervention measures were developed and tested in the experimental study sites during the intervention phase and their role in improved participation and access to MDA assessed during the post-intervention phase. Table 2 presents the barriers, intervention strategies as presented through the county and sub-county stakeholders’ meetings and the community FGDs.

**Table 2:** Barriers to MDA access and tested interventions/strategies.

	Identified Barrier	Source	Tested Intervention/Strategy
1.	Limited knowledge on LF and need to take drugs to interrupt transmission of infection	- County and Sub-county stakeholders’ meetings -Community FGDs	-Number of Health education materials distributed increased- posters, banners in all public places and brochures, at least one per household to dispel myths about cause of LF -Increased period from one to two weeks on Health education regarding the diseases, its causes, prevention and susceptibility
2.	Limited awareness of community members on drugs used, their benefits and side effects, method of distribution, eligibility and reasons for repeated annual rounds	- County and Sub-county stakeholders’ meetings -Community FGDs	**-** Support in awareness creation by the health workers -Increased awareness creation period with repeated messages about drugs used, benefits and side effects, eligibility and why the repeated annual rounds -Involving village elders in awareness creation
3.	Inadequate training of CHEWs and selected CDDs limiting their responses to questions from the community members, poor record-keeping and failure to directly observe treatment by CDDs	-County and Sub-county stakeholders’ meetings	**-**Meetings with CHEWs and CDDs to answer questions on unclear issues arising from the trainings -Emphasis on importance of Directly Observed Treatment (DOT)
4.	Failure to revisit persons missed on initial visits by CDDs to maximize coverage	-County and Sub-county stakeholders’ meetings -Community FGDs	**-**Emphasis to CDDS on importance of revisits and need to have a daily schedule of drug distribution that is based on community members’ availability since the CDDs know the drug recipients who live in their neighborhood -Allocation of CDDs target number of persons to treat based on vastness (distance between one household and the other) of the area as opposed to number of households and people
5.	Failure to adhere to CDDs selection criteria resulting in some CDDs being too senior in age, unknown to the community members, not having gone to school	-Community members FGDs	**-**Replace the older CDDs with new younger ones* - Select CDDs who have attained basic education level **-**Emphasis on importance of assigning the CDDs to the areas where they come from and are known by the community *
6.	Failure of CDDs to observe hygiene during drug administration	-Community FGDs	Encouraging CDDs to avoid touching the drugs with fingers during administration and make use of plastic spoons provided

*represents intervention strategies that the study could not implement directly but recommends to the Programme Implementers

### Socio-demographic factors and their association with access to LF drugs

Table 3 outlines the association between socio-demographic factors and access to LF drugs.

From the results, male participants were significantly more likely to access the drugs compared to female participants, (OR = 1.94, p = 0.008). Older participants aged above 30 years (OR = 1.78, p = 0.354) showed higher likelihood to access drugs, although the associations were not significant. Similarly, participants who were currently married (OR = 1.67, p = 0.137) or widowed, divorced or separated (OR = 1.17, p = 0.719) had a higher likelihood to access the drugs than those who were single, although the association was not significant.

**Table 3:** Association between socio-demographic factors and access to LF drugs.

Factors	N = 688 n (%)	Likelihood of MDA access [OR (95%CI)]	p-value
**Socio-demographic factors**			
**Gender:**			
Male	255 (37.1%)	1.94 (0.19–3.16)	0.008*
Female	433 (62.9%)	Reference	
**Age group (years):**			
<20	21 (3.1%)	Reference	
20–30	170 (24.7%)	0.73 (0.23–2.37)	0.605
30–40	161 (23.4%)	1.78 (0.53–6.01)	0.354
40–50	119 (17.3%)	1.93 (0.55–6.78)	0.303
50–60	99 (14.5%)	1.27 (0.37–4.37)	0.707
>60	118 (17.2%)	1.65 (0.47–5.76)	0.430
**Marital status:**			
Single	76 (11.1%)	Reference	
Currently married	533 (77.5%)	1.67 (0.85–3.29)	0.137
Widowed/Divorced/Separated	79 (11.5%)	1.17 (0.50–2.70)	0.719
**Religion:**			
Christian	464 (67.4%)	Reference	
Islam	24.0%	1.07 (0.64–1.79)	0.800
Non-practicing	57 (8.3%)	1.78 (0.68–4.68)	0.242

* Indicates a statistically significant association (p-value < 0.05)

### Socio-economic factors and their association with access to LF drugs

The main occupation was significantly associated with access to MDA; participants whose main occupation was either farming or fishing were more likely to access MDA compared to those in other occupations (OR = 2.79, p = 0.012). Similarly, participants who engaged in casual labor as their main occupation were significantly more likely to access MDA than those in other occupations (OR = 3.02, p = 0.021).

The type of toilet facility owned was significantly associated with access to MDA; participants who owned a traditional pit latrine were more likely to access MDA than those who owned a flush-type of toilet (OR = 2.87, p = 0.002). The type of water source used was also significantly associated with access to MDA; participants who used improved water source were less likely to access MDA than those using unimproved water source (OR = 0.54, p = 0.005) (Table 4).

**Table 4:** Association between socio-economic factors and access to LF drugs.

Factors	N = 688 n (%)	Likelihood of MDA access [OR (95%CI)]	p-value
**Socio-economic factors**			
Education:			
No education	240 (34.9%)	Reference	
Primary	332 (48.3%)	1.16 (0.73–1.85)	0.531
Secondary	101 (14.7%)	1.26 (0.63–2.52)	0.510
Post-secondary	15 (2.2%)	2.24 (0.27–18.18)	0.452
Occupation:			
Business (large)	124 (18.0%)	2.28 (0.93–5.61)	0.073
Housewife	109 (15.8%)	1.99 (0.83–4.80)	0.125
Salaried worker	10 (1.5%)	1.83 (0.32–10.36)	0.493
Farmer/Fisherman	291 (42.3%)	2.79 (1.25–6.25)	0.012*
Casual laborer	109 (15.8%)	3.02 (1.18–7.72)	0.021*
Other occupations	45 (6.5%)	Reference	
Toilet facility:			
No toilet	141 (20.5%)	0.84 (0.50–1.42)	0.523
Flush toilet	66 (12.1%)	Reference	
Traditional pit latrine	454 (83.0%)	2.87 (1.48–5.59)	0.002*
VIP latrine	66 (12.1%)	1.65 (0.51–5.33)	0.401
Roof material:			
Thatch/Palm leaf/Makuti	148 (21.5%)	0.94 (0.56–1.58)	0.820
Iron sheet	539 (78.3%)	Reference	
Floor material:			
Earth/Mud/Dung/Sand	521 (75.7%)	Reference	
Wood planks	5 (0.7%)	0.42 (0.04–4.64)	0.476
Palm/Bamboo	16 (2.3%)	Omitted	
Polished wood	1 (0.2%)	Omitted	
Ceramic tiles	11 (1.6%)	1.66 (0.20–13.49)	0.634
Cement	130 (18.9%)	0.65 (0.38–1.11)	0.112
Carpet	4 (0.6%)	0.10 (0.01–1.16)	0.066
Wall material:			
Cane/Palm/Trunks	2 (0.3%)	Omitted	
Cement	54 (7.9%)	0.84 (0.37–1.91)	0.675
Bamboo with mud	82 (11.9%)	16.76 (2.29–122.80)	0.006*
Mud/Dung	415 (60.3%)	Reference	
Stone/Cement with mud	23 (3.3%)	0.22 (0.08–0.62)	0.004*
Stone with cement	72 (10.5%)	0.94 (0.45–1.97)	0.866
Bricks with cement	28 (4.1%)	0.61 (0.24–1.50)	0.281
Blocks with cement	8 (1.2%)	0.34 (0.05–2.05)	0.236
Iron sheet	4 (0.6%)	0.22 (0.03–1.62)	0.138
Cooking fuel:			
Firewood	597 (86.8%)	Reference	
Charcoal	80 (11.6%)	0.77 (0.40–1.49)	0.444
Kerosene/Paraffin	3 (0.4%)	Omitted	
Gas	7 (1.0%)	Omitted	
Water source:			
Unimproved	348 (50.6%)	Reference	
Improved	340 (49.4%)	0.54 (0.35–0.83)	0.005*

* Indicates a statistically significant association (p-value < 0.05)

### Respondents’ knowledge about clinical symptoms of lymphatic filariasis during the pre-intervention and the post-intervention phase

The results showed a significant increase in study respondents’ knowledge of people with lymphedema (p<0.001). During the pre-intervention phase; about a quarter (25.1%) of the respondents reported knowing someone with swollen limbs (lymphoedema), and on average they knew at least one person with clinical symptoms (range: 1–11 persons). After implementation of the interventions, 43.7% of the respondents in the experimental sites compared to 3.5% in the control sites stated that they knew someone with lymphoedema.

Similarly, the study results showed that there was a significant increase in participants’ knowledge of people with hydrocele (p = 0.006). During the pre-intervention phase, more than half (57.3%) of the respondents reported knowing at least one person with clinical symptoms of hydrocele (range: 1–5 persons). After implementation of the interventions, 57.5% of the respondents in the experimental sites compared to 38.8% in the control sites reported knowing someone with hydrocele (Table 5).

**Table 5:** Respondents’ knowledge about clinical symptoms of lymphatic filariasis.

Outcomes	Pre-Intervention (n = 350)		Post-Intervention (n = 338)		Difference between control and experimental groups during post-intervention	Overall (n = 688)
	Control (n = 173)	Experimental (n = 177)	Control (n = 170)	Experimental (n = 168)		
# of households	173	177	170	168	-	688
Proportion of participants who know someone with lymphoedema	24 (13.9%)	64 (36.2%)	6 (3.5%)	73 (43.7%)	Diff = 40.2, p < 0.001*	167 (24.3%)
How many people with lymphoedema do you know? [mean; range]	1.2 (1–3)	1.7 (1–11)	1.8 (1–6)	1.2 (1–5)	-	1.4 (1–11)
Proportion of participants who know someone with hydrocele	90 (52.0%)	110 (62.2%)	66 (38.8%)	96 (57.5%)	Diff = 18.7, p = 0.006*	363 (52.8%)
How many people with hydrocele do you know? [mean; range]	1.5 (1–4)	1.5 (1–5)	1.9 (1–10)	1.4 (1–4)	-	1.6 (1–10)
Proportion of participants who do not know that they are at risk of either lymphoedema or hydrocele	78 (45.1%)	90 (50.9%)	88 (51.8%)	75 (44.9%)	Diff = 6.9, p = 0.2044	332 (48.3%)

*Indicates a statistically significant difference between control and experiment groups

**Fig 2:**
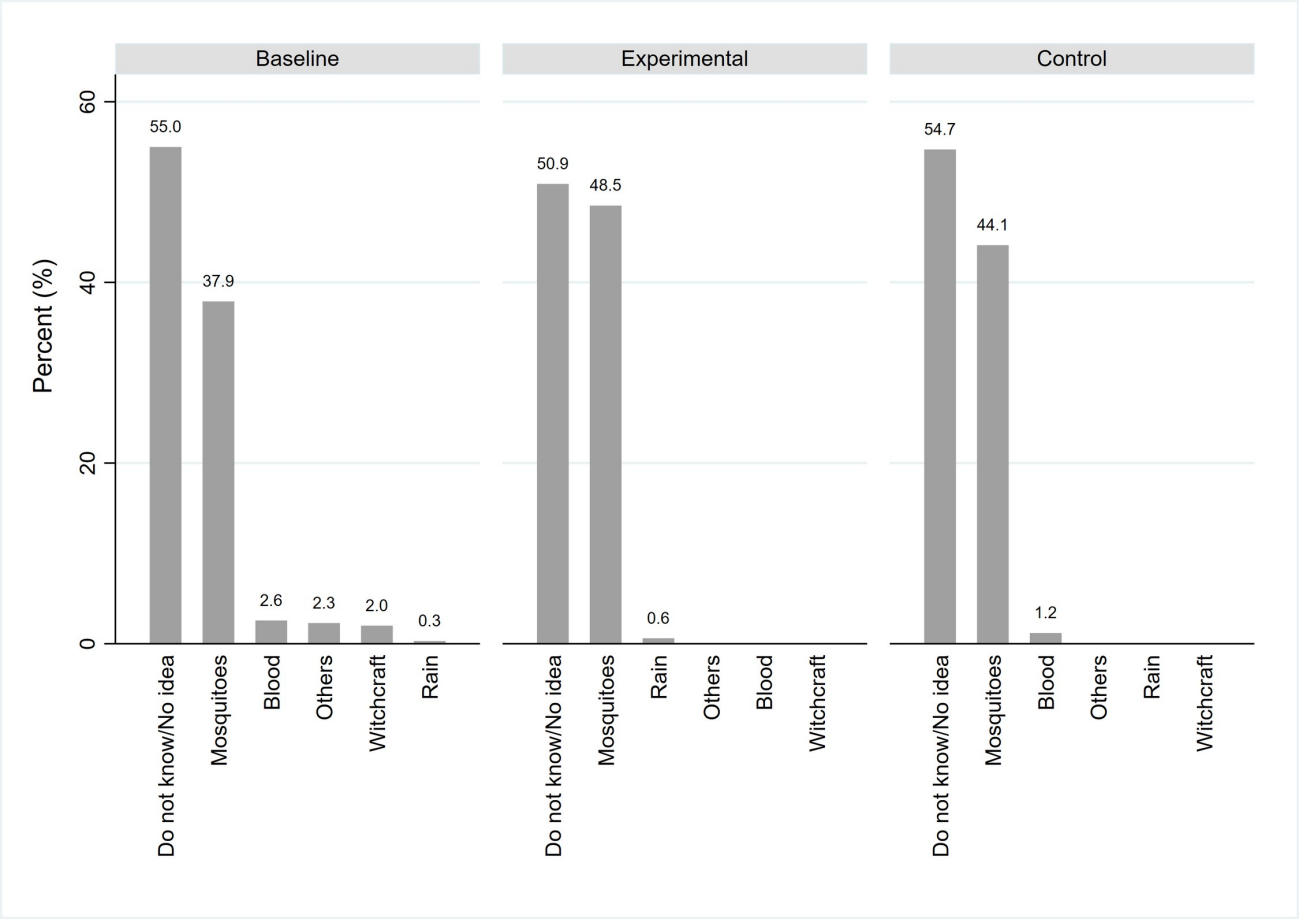
Reported causes of lymphoedema.

However, just over half (55%) of the respondents in the pre-intervention phase did not know the cause of lymphoedema, and only 37.9% accurately stated that it is caused through mosquito bites (Fig 2). After implementation of the interventions a higher proportion, 48.5% of those in the experimental group accurately stated that lymphoedema is caused through mosquito bites compared to 44.1% in the control group.

Participants in the FGDs with youth male and adult women in the pre-intervention phase stated that lymphoedema is caused by:

“Some even believe in witchcraft. With swollen limbs it can be said the infected stepped on something evil.” (FGD-KLN-CM-YM-005)

“Hygiene is very important; everybody can get swollen limbs as long as he/she does not keep hygiene.” (FGD-KYG-CM-AW-004)

Furthermore, a majority, 61.5% of the respondents in the household survey did not know the cause of hydrocele, and only 31.3% accurately stated that it is caused through mosquito bites.

Respondents of the FGDs with adult women in the pre-intervention phase stated that:

“It’s caused by frequently riding a bicycle and motorbikes, also if one has stomach aches, for instance, my father used to carry charcoal from far in the forest to town then he developed stomach aches and later Filariasis” (FGD-KLN-CM-AW-003)

**Fig 3:**
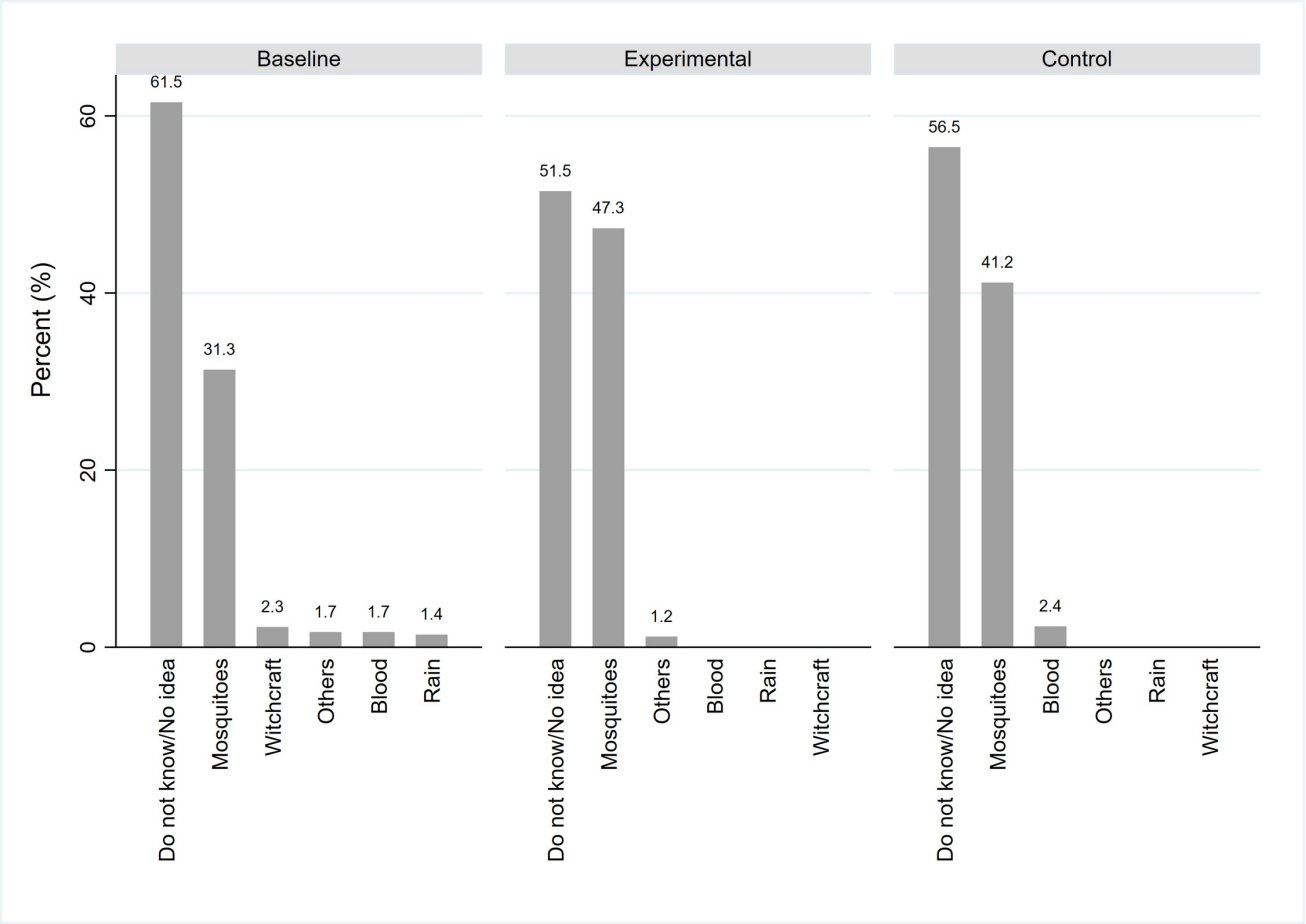
Reported causes of hydrocele.

After implementation of the interventions; over half (57.5%) of the participants in the experimental group reported knowing someone with hydrocele compared to only 38.8% in the control group, with more, 47.3% of the participants in the experimental group accurately stating that hydrocele is caused through mosquito bites compared to 41.2% in the control group (Fig 3).

It was however observed in the pre-intervention phase that 2.0% and 2.3% of all the respondents indicated that lymphoedema and hydrocele respectively are caused by witchcraft. However, the number of participants having this perception dropped to zero during post-intervention survey.

The gap in knowledge of LF disease was further observed during the post-intervention phase where a majority of the participants in the FGDs with adult men stated that:

“One person having an affair with one who has swollen genitals might get the disease through intercourse and that’s it.” (FGD-KYG-CM-AM-003)

“The existence of the baobab tree in the community. People living in areas with the baobab tree have swollen limbs to represent the nature of the tree.” (FG-KYG-CM-AM-004)

### Respondents’ perceived risk of susceptibility of infection with lymphatic lilariasis during the pre-intervention and the post-intervention phase

Overall, 48.3% of the respondents did not know that they were at risk of getting either lymphoedema or hydrocele, and 20.8% were categorical that they are not at risk of LF infection. About a half, 51.8% of the participants who did not know that they were at risk were in the control group. However, the difference in proportions of participants who did not know that they were at risk was not statistically different between the control and experimental groups (p = 0.2044).

Participants in the FGDs with youth male in the pre-intervention phase stated that:

“Some say it is inherited from the bloodline (genetics). For example, if your grandfather had hydrocele then it is no surprise the rest of the family might get infected.” (FGD-KLN-CM-YM-005)

Notably, during the post- intervention phase participants in the adult men and youth female groups still showed a lack of awareness of risk susceptibility and stated that:

“The awareness of the swollen genitals was not that good I just saw the CDDs at my place though I took the drugs.” (FGD-KYG-CM-AM-003)

“Too much cooking oil. If you use too much cooking oil it might cause that problem.” (FGD-KLN-CM-YF-003)

### Uptake of LF drugs and respondents’ perceptions on treatment pre and post-intervention phases

During the pre-intervention phase, less than three quarters 247 (70.6%) of the participants reported having ever taken LF drugs with 199 (80.6%) of them reporting that they had taken the drugs during the previous MDA. The surveyed participants had on average taken LF drugs for two times (years) (range: 1–7 times). After implementation of the interventions, the proportion of participants who took drugs during the previous MDA insignificantly rose to 82.9% (Diff = 2.3, p-value = 0.4351).

After implementation of the interventions; the proportion of participants who considered the treatment as necessary significantly increased to 96.2% from 88.3% during pre-intervention, phase and the proportion of participants who expressed problems with swallowing drugs due to size, number, or taste of drugs all significantly dropped. Reduced proportion of respondents 18 (5.3%) during post-intervention phase affirmed that they would not take the drugs again mainly due to the fear of the reactions or side effects (42.1%) or they considered the drugs as not necessary for them (26.3%) (Table 6).

**Table 6:** Uptake of LF drugs and respondents’ perceptions on treatment.

Outcomes	Pre-interventions (n = 350)	Post-interventions (n = 338)	Difference between pre- and post-interventions	Overall (n = 688)
Proportion ever taken LF drugs	247 (70.6%)	316 (93.5%)	Diff = 22.9, p < 0.001*	563 (81.8%)
Proportion who took LF drugs during last MDA	199 (80.6%)	262 (82.9%)	Diff = 2.3, p = 0.4351	461 (81.9)
Number of times taken LF drugs [mean; range]	2.1 (1–7)	2.4 (1–6)	-	2.3 (1–7)
Proportion who consider treatment as necessary	309 (88.3)	325 (96.2)	Diff = 7.9, p = 0.001*	634 (92.2)
Proportion who expressed problem swallowing drugs	43 (12.3%)	10 (3.0%)	Diff = 9.3, p < 0.001*	53 (7.7%)
Proportion who expressed problem with size of drugs	29 (8.3%)	4 (1.2%)	Diff = 7.1, p < 0.001*	33 (4.8%)
Proportion who expressed problem with number of drugs	12 (3.4%)	1 (0.3%)	Diff = 3.1, p = 0.027*	13 (1.9%)
Proportion who expressed problem with taste of drugs	30 (8.6%)	6 (1.8%)	Diff = 6.8, p = 0.001*	36 (5.2%)
Proportion who affirmed to take LF drugs again	297 (84.9%)	320 (94.7%)	Diff = 9.8, p < 0.001*	617 (94.7%)

*Indicates a statistically significant difference between pre- and post-intervention groups

Those who had never taken drugs majorly argued that they are not infected with LF and thus there was no need to take the drugs (60.4%), always away on work during drug distribution (30.2%), never reached by the CDDs (12.4%), fear of side effects (10.5%). The CDDs are instructed not to administer the drugs to pregnant women but to treat those who are 2 weeks post-delivery and 2.3% of the respondents gave being pregnant as a reason for not having ever taken the LF drugs.

Participants in the FGDs with community members in the pre- as well as the post-intervention phase further stated that:

“I have never taken them. Because let’s be frank you cannot take something that you don’t know where it comes from and you are not told about it. You are just expected to take the drugs like that. I cannot take something that I don’t know. Due to lack of awareness, we thought the drugs were for trials thus we refused to take them.” (FGD-KLN-CM-YM-006)

“Those who bring those drugs, they should tell us about the side effects as well, I think if possible they should be given at night so that we sleep since it will be already night. Let them educate us first and people shall accept the drugs” (FGD-KYG-CM-AW-001)

“People should be made aware about the drugs and made to understand what the drugs are for because some refuse to take the drugs because they do not know what they are for.” (FGD-KLN-CM-YF-003)

“I would like to contribute on that, the community needs to be informed more on first, the disease itself, secondly, on the dangers of the disease.” (FGD-KLN-CM-YF-007)

### Respondents’ knowledge about mass drug administration

During the pre-intervention phase, 88.6% of the respondents reported that they had heard about MDA for elimination of LF in their community. After implementation of the interventions, a larger proportion, 92.0% reported having heard about MDA, the majority of these people were in the control group 167 (98.2%). In the post-intervention phase, the proportion of participants who had heard about the MDA was significantly more in the control group than the experimental group (p < 0.001) (Table 7).

**Table 7:** Respondents’ knowledge about mass drug administration.

Outcomes	Pre-Intervention		Post-Intervention		Difference between control and experimental groups during post-intervention	Overall (n = 688)
	Control (n = 173)	Experimental (n = 177)	Control (n = 170)	Experimental (n = 168)		
Number of households	173	177	170	168	-	688
Proportion of participants who have heard about MDA	164 (94.8%)	146 (82.5%)	167 (98.2%)	144 (85.6%)	Diff = 12.6, p < 0.001*	621 (90.3%)
Most common channel through which they heard about MDA	CDDs & chief’s meeting	CDDs & friends	CDDs & Radio campaigns	CDDs & Radio campaigns	-	CDDs & Radio campaigns
Proportion of participants reached by this channel	116 (72.0%)	72 (49.7%)	148 (88.1%)	71 (41.8%)	Diff = 46.3 p < 0.001*	407 (51.5%)

*Indicates significant difference between control and experiment group

Participants in the FGDs with community members stated that:

“People should get informed on the exact days when the distribution will be done so that they may stay at their house on those specific days.” (FGD-KLN-CM-YF-003)

“At times they inform you, as they give you the drugs, for me I can’t accept that, I think we need time for awareness first, then we decide before the MDA day” (FGD-KLN-CM-YM-006)

In an FGD with youth male community members, a participant indicated that he has never taken the drugs stating that not being well sensitized and prepared made him refuse to consume the drugs during the previous MDA.

“I have never taken the drugs but in the community some consumed them, while others refused. For me I refused because I was ambushed.” (FGD-KLN-CM-YM-006)

A youth female participant further indicated that information on drug distribution gets to the community when the distribution has already started.

“We suggest that someone tell us about drug distribution before it is done but we are getting that information while the exercise is going on.” (FGD-KYG-CM-YF-002)

### Source of information on MDA

**Fig 4:**
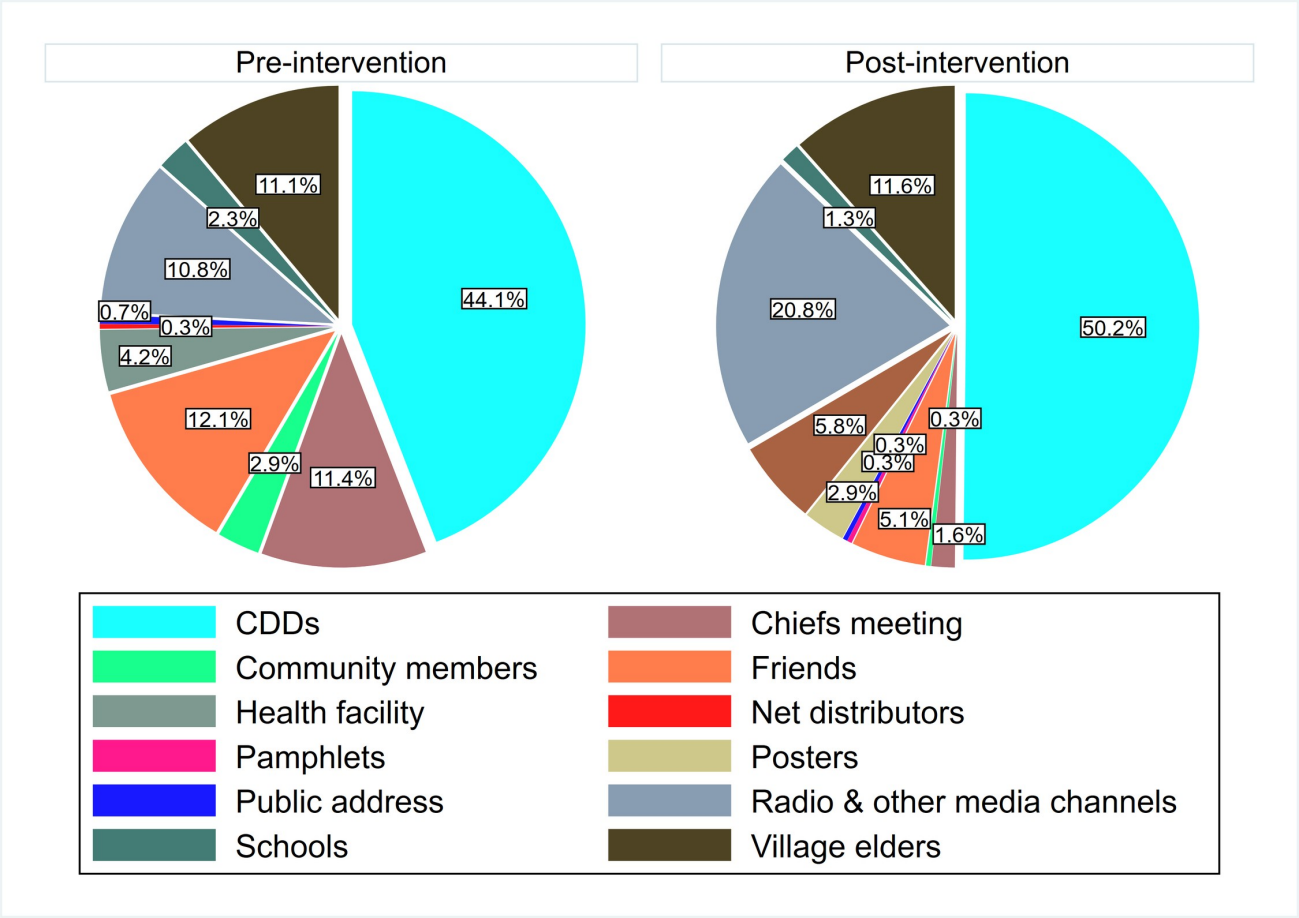
Sources of information about MDA.

Fig 4 shows the proportion of study participants reached with information about MDA through various channels during pre- and post- intervention phases. More people were reached with information either through CDDs or radio and other media channels during post-intervention compared to pre-intervention phase.

The information given regarding MDA includes; distribution method, administration dates, eligibility criteria, importance of taking the drugs, and how to take the drugs. However, about 9.7% of the respondents reported not receiving any information about MDA. The participants reported receiving varied information about possible side effects of the drugs as dizziness, nausea, and vomiting.

In overall, over half of the respondents acknowledged receiving this information at least once a year (66.2%), a quarter of the respondents reported receiving the information only once in their lifetime or very rarely (25.0%). However, 3.5% of the respondents could not remember receiving the information.

Regarding awareness creation and distribution time, participants in the FGDs stated that:

“I think they should increase the awareness creation time. Let the information be availed early enough rather than people being ambushed” (FGD-KLN-CM-YF-008)

“They should increase the MDA duration in terms of the awareness time and distribution time. Those people from far usually just see the CDDs with the drugs.” (FGD-KYG-CM-AW-001)

“No the period is not enough, not everyone gets the information. People usually just see the CDDs on their doorsteps without informing them.” (FGD-KLN-CM-YM-005)

## Discussion

The results reported in this paper indicate that certain socio-demographic factors; gender and age influenced community participation in MDA for LF. Male community members accessed the drugs more than their female counterparts. Females have been reported to frequently present lower rates of compliance with MDA [25] and a study conducted in Brazil indicated the need for a differentiated approach towards the female population in order to achieve more successful reach [26]. Reasons cited for lack of access to treatment in similar studies included pregnancy, lactation, lack of information and fear of spontaneous abortion, along with domestic situations in which women are prohibited from participating in MDA because of their husbands’ negative beliefs about it [27, 28]. Pregnant women and breast feeding mothers are usually excluded from the MDA program in Kenya [29].

Furthermore, women who are housewives may not have the freedom to make decisions on when to receive medical services without the consent of their husbands who most likely would be away fending for their families at the time of the CDDs’ visit to the household. Addressing gender disparities to ensure that no one is left behind in the fight against LF is an important factor for consideration as described in a study conducted in Ethiopia [30].

The current study results also show that age influenced community participation in MDA. Older persons, 30 years and above accessed the treatment more than their younger counterparts. Similar results have been reported previously [27, 18]. It can be argued that older persons are usually more exposed to health programmes and in most cases have realized the benefits of participating in such campaigns.

The results of the current study have also shown that some socio-economic factors influenced participation in MDA. Persons whose main occupation is fishing, farming and casual laborer who are considered to be of low socio-economic status and ready to take free health services accessed the drugs more as compared to salaried workers and large business owners who possibly feel that they are able to pay for health services [31]. Furthermore, community members from households that use pit latrines and draw water from unimproved water sources considered to be of low socio-economic status participated in MDA more than those that use improved water source and other types of toilets and considered to be of high socio-economic status.

Results of the current study showed that there were low levels of knowledge of the disease among the community members. In general, people with low knowledge usually also give low priority to disease prevention [29]. The developed and tested strategies were impactful in increasing knowledge on LF disease and the study suggests planned health education and repeated awareness creation for behavior change communication on the cause of the disease and the importance of prevention of infection. These need be conducted more frequently and not left to the period of MDA campaigns solely. Providing information regarding disease transmission factors can increase community participation in MDA as reported in similar studies [32, 33].

The current study results showed an increase in the proportion of participants who took the drugs as well as those who considered the treatment to be necessary after the implementation of the interventions. Observed improvements in coverage have been reported to be associated with implementation of strategies [34]. Moreover, the current study has shown a decrease in the proportion of those who had problems with the number, size and taste of the drugs. The study results have further shown a reduction in the proportion of those who feared taking the drugs due to side effects. These changes could be attributed to the interventions that were implemented in the study. As reported in a study conducted in India, provision of information on the side effects and benefits of the treatment as well as the schedule of distribution is instrumental in increasing participation in the programme [35]. Evidence from multiple settings indicates that individual participation in public health programs is limited by lack of information [36].

Results of the current study have further shown an increase in the proportion of the participants who had heard about MDA in the post-intervention phase compared to the pre-intervention phase. A majority of the participants in both phases of the study indicated that they got the MDA information from the CDDs. There is a need to therefore invest in the CDDs training to improve their efficiency in carrying out their roles. Inadequately trained volunteers are known to get intimidated when unable to confidently answer questions posed by community members [37]. Motivation of the CDDs is also vital if they should be expected to serve in multiple roles which include health education about the disease, awareness creation on the MDA and the actual drug distribution and record keeping. The CDDs selection criteria ought to be strictly adhered to as it clearly has an influence on community participation in MDA for LF [38, 39]. The results have also shown the importance of ensuring that enough time is given for community members to be empowered with information and be prepared to receive the treatment which is key to the success of the campaign and has also been reported in a study conducted in Pekalongan [40].

## Study Limitations

This study had a limitation of not effectively controlling for the spill over effects of the movement of participants on their normal daily activities between the control and the experimental groups. This limitation contributed to the instances where the observed differences in the outcome of interest between the control and experimental groups were statistically insignificant.

## Conclusion

This study demonstrated some of the barriers to community participation in MDA and strategies that can be used to overcome them for improved programme performance. Community participation in addressing the barriers is key to the success of the MDA. Cleary, better approaches are needed to educate the community members on LF disease and the importance of prevention of infection. There is need to plan and implement awareness creation strategies with emphasis on the drugs used, their safety, action and eligibility criteria. Since MDA occurs once a year and heavily engages most components of the health system, there is need to ensure robustness of such components for the program to improve its reach to the community members with special considerations for both genders in all treatment rounds.

## Supporting information

S1 Text Appendix 1 Household Survey Questionnaire. (DOC)

S2 Text Appendix 2 FGD Guide. (DOC)

S3 Text Appendix 3 kilifi stakeholders feedback meeting. (DOC)

S4 Text Appendix 4 kaloleni stakeholders feedback meeting. (DOC)
