# Supplementary material for: Addressing barriers of community participation and access to mass drug administration for lymphatic filariasis elimination in Coastal Kenya using a participatory approach
Source: PLoS Negl Trop Dis. 2020 Sep 16;14(9):e0008499. doi: 10.1371/journal.pntd.0008499 (PMC7494106; doi:10.1371/journal.pntd.0008499)
Supplement: S1 Text — (DOC) [file pntd.0008499.s001.doc]

### S1_Text.doc

### Appendix 1: Interview schedules for heads of households (male or female)

Date: _____________________

ID: __________________________

Sub-County: ______________________

Ward: ______________________________________

Household no: ________________________________

| Instructions:   - This form should be used for interviews to Household heads. - If the participant refuses to answer a question, circle the number of the question and do not mark any answers for that question. - After obtaining informed consent, read the following instructions to the participant: |
| --- |
| **“I am going to ask you questions about the Filariasis control program, whether you have been treated by the programme, your willingness to participate by taking the drugs and preferences for being reached during treatment. Some questions about your household members focusing on the control program will be asked to you. Please answer the questions as honestly as you can remember. Your information will be kept private and this form will not have your name anywhere, you will be identified by a number only. If you have any questions or do not understand what I am asking you at any time, please ask for clarification. Some questions may prove embarrassing to you.**  **Please remember that you do not have to answer any questions that you do not want to answer and you may discontinue the interview at any time. Do you have any questions before we begin?”** |

**Socio-Demographic** **Characteristics**

1. Sex (Tick) Male ( 1) Female ( 2)

2. Age in Years_______________________

3. Marital Status (Tick)

Single (1)

Currently Married (2)

Divorced/Widowed (3)

4. Religion (Tick)

Christian (1)

Islam (2)

Non-practicing (3)

Others, specify (4) __________________

**Socio-economic characteristics**

5. Level of Education (Tick)

Never attended school (1)

Did not complete primary school (2)

Completed primary school but did not complete secondary school (3)

Completed secondary school (4)

Further studies after secondary school (5)

Others, specify (6) _____________________

6. Main occupation (Tick)

Small business (kiosk, kibanda, motorcycle operator) (1)

Big business (shop) (2)

Housewife (3)

Salaried worker (teacher, police, chief) (4)

Fisherman (5)

Farmer (6)

Casual laborer (7)

Others, specify (8) _____________________

7. Presence of toilet facility in the home (Tick) Yes (1) No (2)

7a, If yes in 7, state type

Flush (1)

Traditional pit latrine (2)

Ventilation Improved Pit (VIP) latrine (3)

No facility, bush, field (4)

Others, specify (5) ___________________

b, Share toilet with other households (Tick) Yes (1) No ( 2 )

8. Type of roofing material of the main house (Tick)

Grass thatch, makuti *(1)*

Tin cans (2)

Corrugated iron sheet (3)

Brick/gall sheet (4)

Concrete (5)

Tiles (6)

Others, specify (7) _______________

9. Type of main flooring material (Tick)

Earth, mud, dung, sand (1)

Wood planks (2)

Palm, bamboo (3)

Polished wood (4)

Ceramic tiles (5)

Cement (6)

Carpet (7)

Others, specify (8) ________________

10, Type of cooking fuel (Tick all that apply)

Electricity (1)

Gas (2)

Kerosene (3)

Charcoal (4)

Firewood straw (5)

Dung (6)

Others, specify (7) _________________

11. Sources of drinking water (Tick all that apply)

Piped into dwelling (1)

Piped into compound/plot (2)

Public tap (3)

Open well in compound/plot (4)

Covered public well (5)

Spring (6)

River, stream (7)

Pond, lake (8)

Dam (9)

Rainwater (10)

Bottled water (11)

Others, specify (12) ____________________

12. Time to water source (Tick)

Less than 15 minutes (1)

More than 15 minutes (2)

13. Water availability (Tick)

Usually available (1)

Several hours per day (2)

Once or twice per week (3)

Infrequent (4)

14. Household owns structure (Tick)

Owns (1)

Pays rent, lease (2)

No rent, with consent of owners (3)

No rent, squatting (4)

Others, specify (5) ________________________

15. Household owns land on which structure sits (Tick)

Owns (1)

Pays rent, lease (2)

No rent, with owner’s consent (3)

No rent, squatting (4)

Others, specify (5) _________________________

16. State of repair of dwelling (Tick)

Completely dilapidated shack (1)

Needs major repairs (2)

Being repaired (3)

Under construction (4)

Others, specify (6) _______________________

17. How household disposes of kitchen waste and trash (Tick)

Regular collection by government (1)

Infrequent collection by government (2)

Pays for private collection (3)

Composted (4)

Dumps, buries, burns in compound (5)

Dumps in street empty plot (6)

Others, specify (7) __________________________

18. Possession of durable consumer goods (Tick)

Radio (1)

Television (2)

Refrigerator (3)

Bicycle (4)

Motorcycle (5)

Car/truck (6)

Solar power (7)

**Questions on knowledge about LF**

19. Do you know of anyone in your community who has swollen limbs (lymphoedema?) (Tick)

1. Yes (1)
2. No (2)

20. How many such people do you know of _______________ (Record a number)

21 What do you think are the causes of swollen limbs (Tick)

1. Witchcraft (1)
2. Rain (2)
3. Blood (3)
4. Mosquitoes (4)
5. Others, specify (5) ___________
6. Do not know/ no idea (6)

22. Do you know of anyone in your community who has swollen genitals (hydrocele)? (Tick)

1. Yes (1)
2. No (2)

23. How many such people do you know of_______________ (record a number)

24. What do you think is the cause of swollen genitals? (Tick)

1. Witchcraft (1)
2. Rain (2)
3. Blood (3)
4. Mosquitoes (4)
5. Others, specify (5) __________
6. Do not know/ no idea (6)

25. Do you consider yourself to be at risk of getting swollen genitals? (Tick)

1. Yes (1)
2. No (2)
3. Do not know (3)

**Questions on knowledge about MDA**

26. Have you heard about Mass Drug Administration for Elimination of Lymphatic

Filariasis in your community? (Tick)

1. Yes (1)
2. No (2)

26a, If yes in Q26,

How did you learn about the MDA? ____________________________________

_________________________________________________________________

What information did you get about the MDA? ____________________________

__________________________________________________________________

__________________________________________________________________

How frequently do you receive this information in one year? _________________

What is your opinion of this source of information? ________________________

___________________________________________________________________________________________________________________________________________________________

26b. What information were you given about the treatment regimen?

__________________________________________________________________

About eligibility for treatment_________________________________________

__________________________________________________________________

About the potential side effects_________________________________________

__________________________________________________________________

26c. How would you want awareness creation to be conducted during the next round?

About materials/strategies_________________________________________________

About duration___________________________________________________________

About the persons creating the awareness_____________________________________

**Questions on Drug use**

27. Did you take drugs during the last MDA (2017)? (Tick)

1. Yes (1)
2. No (2)
3. Cannot remember (3)

28a. How many times have you taken the LF drugs during MDA? _______________

28b. If you have never taken LF drugs during MDA, give your reasons for not taking.

________________________________________________________________________________________________________________________________________________________________________________________________________________________________________________________________________________________________________________________

**Questions on perceptions of the treatment**

29. Do you consider this treatment as necessary for you? (Tick)

1. Yes (1)
2. No (2)

30. Do you have trouble swallowing the drugs? (Tick)

1. Yes (1)
2. No (2)

31. Do you have a problem with the size of the drugs given to you? (Tick)

1. Yes
2. No

32b. If yes, specify the problem __________________________________________

32 c. Do you have a problem with the number of the drugs given to you? (Tick)

1. Yes
2. No

32d. If yes, specify the problem __________________________________________

32e. Do you have a problem with the taste of the drugs given to you? (Tick)

1. Yes
2. No

32f. If yes, specify the problem __________________________________________

33. Would you be interested in taking these drugs next time? (Tick)

1. Yes (1)
2. No (2)
3. Do not know (3)

34. If no, why not? (Tick as many as applicable)

- - They were not distributed in my house or village (1)
  - Was absent on the day they were distributed (2)
  - Do not like modern medicine (3)
  - Is not necessary for me (4)
  - Fear of reactions/ complications (5)
  - Have another illness (6)
  - Others, specify (7) __________________________

35. Next time the drugs are administered; would you want them to be distributed the same way as this last time? (Tick)

- - 1. Yes (1)
    2. No (2)
    3. Do not know (3)

36. If no, why not? (Tick)

- Had to wait for CDD for long hours (1)
- CDD did not explain well the need to take the drug and its side effects (2)
- CDD did not have enough drugs to give (3)
- Poor interaction with the CDD (4)
- Others, specify (5) _____________________

37. How would you prefer the next MDA to be conducted?

Distribution method: ______________________________________________________

Distributors: _____________________________________________________________

Distribution duration: ______________________________________________________

Do you have anything else to say?

**THANK YOU VERY MUCH FOR YOUR COOPERATION**
